# HDL-localized plasma ceramides support body temperature regulation

**DOI:** 10.1093/geroni/igac059.1266

**Published:** 2022-12-20

**Authors:** Gina Wade, Judith Simcox

**Affiliations:** University of Wisconsin - Madison, Madison, Wisconsin, United States; University of Wisconsin - Madison, Madison, Wisconsin, United States

## Abstract

As organisms age, the balance of energy expenditure is disrupted. One of the major ways in which this disruption is observed is in an inability to maintain body temperature. Our lab is interested in understanding the signals that regulate this energy balance, and we focus on lipids as energy substrates and signaling molecules to regulate this process. During cold exposure, plasma lipids produced by peripheral tissues are required to fuel and activate heat production in the brown adipose tissue. One of the lipid classes that increases with cold exposure is plasma ceramides. Beyond cold exposure, ceramide lipids are also elevated in aged individuals and are associated with increased cardiovascular disease risk and age-related diseases such as Alzheimer’s disease where they are thought to signal inflammation. However, the functions of ceramides in non-disease states are unknown. To address this gap in knowledge, we have shown that ceramide production in 12-week-old C57Bl6/J mice is required for body temperature maintenance in the cold. Moreover, plasma ceramide levels in 2-year-old mice are unchanged in the cold, and these mice are unable to maintain their body temperature. In disease states, ceramides are transported through the plasma in LDL, but ceramides are enriched in the HDL plasma fraction in the cold. This differential plasma lipid transport suggests dynamic modes of lipid uptake and tissue targeting to regulate energy expenditure. This work will identify molecular mechanisms governing ceramide function in the mammalian response to cold and better our understanding of the systemic lipid metabolism dysregulated in disease.

